# Evaluation of pooled association tests for rare variant identification

**DOI:** 10.1186/1753-6561-5-S9-S118

**Published:** 2011-11-29

**Authors:** Wan-Yu Lin, Boshao Zhang, Nengjun Yi, Guimin Gao, Nianjun Liu

**Affiliations:** 1 Department of Biostatistics, University of Alabama at Birmingham, 1665 University Boulevard, Birmingham, AL 35294, USA; 2 Department of Biostatistics, School of Medicine, Virginia Commonwealth University, Richmond, VA 23298-0032, USA

## Abstract

Genome-wide association studies have successfully identified many common variants associated with complex human diseases. However, a large portion of the remaining heritability cannot be explained by these common variants. Exploring rare variants associated with diseases is now catching more attention. Several methods have been recently proposed for identification of rare variants. Among them, the fixed-threshold, weighted-sum, and variable-threshold methods are effective in combining the information of multiple variants into a functional unit; these approaches are commonly used. We evaluate the performance of these three methods. Based on our analyses of the Genetic Analysis Workshop 17 data, we find that no method is universally better than the others. Furthermore, adjusting for potential covariates can not only increase the true-positive proportions but also reduce the false-positive proportions. Our study concludes that there is no uniformly most powerful test among the three methods we compared (the fixed-threshold, weighted-sum, and variable-threshold methods), and their performances depend on the underlying genetic architecture of a disease.

## Background

In the past several years, genome-wide association studies (GWAS) have successfully identified many common single-nucleotide polymorphisms (SNP) (say, minor allele frequency [MAF] > 5%) associated with complex human diseases. Despite the findings from GWAS, a large portion of the remaining heritability cannot be explained by these common variants [1]. The importance of detecting rare variants has thus been recognized. However, exploring rare variants that are associated with diseases is challenging because of their low frequencies and individually small contributions to the susceptibility to a disease [2]. Recently, several methods have been proposed for detecting rare variants (for an overview see Dering et al. [3]). Most of these methods pool signals of multiple rare variants into a functional unit, such as a candidate gene, and then test the association between the pooled signal and the disease [4-7]. For these methods, the choice of a threshold to discriminate rare variants from common variants plays an important role. If the threshold is too high, variants with relatively high MAFs will dominate the results of association tests for the genes. On the other hand, if the threshold is too low, the statistical power of the association tests will tend to become unnecessarily low. The specification of a threshold is crucial to the performance of a pooled association test. In this paper, we evaluate the performance of several methods using the simulated data of unrelated individuals from Genetic Analysis Workshop 17 (GAW17) [8].

## Methods

### Three analysis methods

Some of the proposed pooling methods first specify a fixed threshold for the MAF and then perform association tests on the set of variants with MAFs smaller than that threshold [4,6]. The weighted-sum method [5] extends this idea and weights each variant by the inverse square root of the expected variance based on the allele frequencies. The larger the MAF, the smaller the weight given to that variant. However, this weighting scheme restricts the effect of a functional variant to be statistically related to its allele frequency, which may not be plausible in some situations. To address the issue of a preset threshold for the MAF, Price et al. [7] proposed a variable-threshold (VT) approach. The VT approach groups rare variants together by searching for an optimal threshold that maximizes the difference between trait distributions for subjects with and without the rare variants. Using the data from GAW17, we compare the performance of the VT method with the performance of the weighted-sum (WS) method [5] and the fixed-threshold method of Morris and Zeggini [6] with thresholds of 1% and 5% (denoted T1 and T5, respectively).

### Data

We use the GAW17 data for 697 unrelated individuals with variants on 22 autosomal chromosomes. There are 24,487 SNPs located in 3,205 genes on these chromosomes. We use the start and end positions (in base pairs) of each gene to pick the SNPs falling within the boundaries of that gene. We analyze all the phenotypes available: Q1, Q2, Q4, and the binary trait (Affected). All the 200 simulated replicates were studied. To evaluate the performance of the four tests (T1, T5, WS, and VT), we requested the simulation answers and compared the answers with the results obtained from the four tests.

### Variable threshold software

All the analyses were performed using the VT test software (http://genetics.bwh.harvard.edu/rare_variants/) of Price et al. [7]. The VT software performs T1, T5, WS, and VT tests in each analysis. The statistical models are all based on linear regressions:(1)

where *y* is the phenotype, *x_i_* is the number of the *i*th rare variant in gene *G*, *w_i_* is the weight given to the *i*th rare variant, and  and  are the estimates of the regression coefficients. For the T1 (or T5) test, *w_i_* equals 1 if the frequency of the *i*th variant is less than 1% (or 5%) and 0 otherwise. For the WS test,(2)

where *p_i_* is the allele frequency of the *i*th variant.

For the VT test, a *z*-score:(3)

is computed for each allele frequency threshold *T*, where SE represents the standard error. Let *z*_max_ be the maximum *z* score among all possible values of *T*; then, the significance of *z*_max_ is assessed by permutation of phenotypes. Suppose that we perform *p* permutations and therefore have *z*_max,1_, *z*_max,2_, …, *z*_max,*p*_, which are the maximum *z* scores obtained at their optimal thresholds *T*_1_, *T*_2_, …, *T_p_*, respectively. To ensure the validity of the VT test, the software allows *T*_1_, *T*_2_, …, *T_p_* for permuted data to be different from the optimal threshold *T* for the original data. The VT software then compares *z*_max_ with *z*_max,1_, *z*_max,2_, …, *z*_max,*p*_ to determine its statistical significance.

When we performed the T1, T5, WS, and VT tests with the VT software, each *p*-value was calculated based on 100,000 permutations. To increase the computation speed, the VT test uses linear regression instead of logistic regression to analyze all phenotypes, including the binary trait. To understand the influence of adjustment for covariates, we compared the results when ignoring all covariates with the results obtained when adjusting for Age and Smoking status. We first obtained the residuals by regressing the phenotypes on Age and Smoking status, and then the residuals were regarded as the adjusted phenotypes and were analyzed by the VT software.

## Results

### Type I error rates

The phenotype Q4 is not related to any of the 3,205 genes, so we use this part of the results to evaluate type I error rates. Given a significance level *α*, we estimate the type I error rate using:(4)

where *I*(·) is the indicator function, *p_i_*_,_*_r_* is the *p*-value of the *i*th gene in the *r*th replicate, *T_g_* is the set formed by all genes, and |*T_g_*| is the number of genes in *T_g_*. Note that the set *T_g_* varies with different methods. Because the T1 test considers only genes that have at least one SNP with MAF less than 1%, the total number of genes considered by the T1 test is 2,485. Similarly, the T5 test considers only genes that include at least one SNP with MAF less than 5%, and the total number of genes considered by the T5 test is 2,881. The WS and VT tests are performed for all genes without prespecifying a threshold, so the total number of genes considered by both of these tests is 3,205.

Table 1 shows the type I error rates for the four tests. When we ignore all the covariates, the type I error rates are generally inflated for the WS and VT tests and slightly inflated for the T5 test. After adjusting for Age and Smoking status, this inflation of type I error rates disappears. We further discovered that the inflation of type I error rates disappeared so long as Age was adjusted, but it remained if only Smoking status was adjusted. To verify this, we deliberately let Age be the outcome variable and tested its association with genes. We found that the rates of rejection of no association (between genes and Age) were generally larger than the nominal significance levels (when the significance level was set at 5%, the average rejection rates were 7.6%, 11.9%, 11.0%, and 12.4% for the T1, T5, WS, and VT tests, respectively). However, when we let Smoking status be the outcome variable and tested its association with genes, the average rejection rates matched the nominal significance levels. This suggests that the observed inflation of type I error rates comes from some latent confounders (e.g., population stratification or preferential death of carriers with some particular genotypes), and we can remove the false-positive findings by adjusting for Age.

**Table 1:** Type I error rates (results based on analyzing Q4)

Significance level	T1	T5	WS	VT
Without adjustment for any covariate				
*α* = 0.05/3,205 = 1.56 × 10^−5^	0.00001	0.00002	0.00055	0.00053
*α* = 0.001	0.00086	0.00272	0.00861	0.00925
*α* = 0.005	0.00468	0.01055	0.02279	0.02384
*α* = 0.01	0.00922	0.01828	0.03454	0.03596
*α* = 0.05	0.04575	0.06324	0.09094	0.09546
*α* = 0.1	0.08971	0.10888	0.13954	0.14894
With adjustment for Age and Smoking status				
*α* = 0.05/3,205 = 1.56 × 10^−5^	0.00001	0.00002	0.00001	0.00001
*α* = 0.001	0.00096	0.00094	0.00095	0.00095
*α* = 0.005	0.00473	0.00491	0.00485	0.00485
*α* = 0.01	0.00956	0.00973	0.00966	0.00972
*α* = 0.05	0.04918	0.05040	0.05037	0.04990
*α* = 0.1	0.09906	0.10148	0.10159	0.10087

### ROC curves

**Figure 1:**
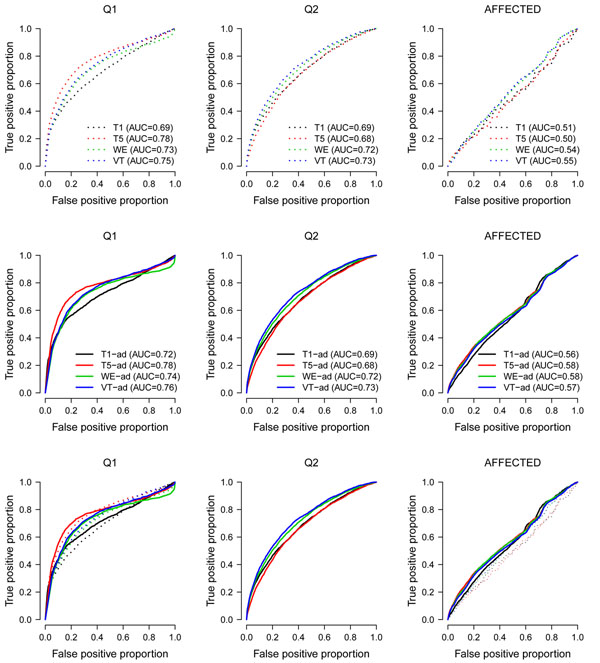
**ROC curves for the four tests**. The top row presents the results without adjustment for any covariate; the second row presents the results with adjustment for Age and Smoking status. In the parentheses are the areas under the ROC curves. In the bottom row we show the combined ROC curves without and with adjustment for covariates.

The phenotypes Q1, Q2, and the binary trait (Affected) are related to some genes, so we use the results on these three phenotypes to evaluate the true-positive proportions and the false-positive proportions. Figure 1 presents the receiver operating characteristic (ROC) curves of the four tests. Given a significance level *α*, we estimate the true-positive proportion using:(5)

and the false-positive proportion using:(6)

where *S_g_* is the set formed by disease genes, |*S_g_*| is the number of genes in *S_g_*, and {*T_g_*\*S_g_*} is the set formed by genes unrelated to the disease. The set of *S_g_* is provided by the underlying simulation model.

From the results, all methods perform better for continuous traits than for the binary trait. For continuous traits, these methods perform better for Q1 than for Q2. This is reasonable because Q1 has higher residual heritability. For phenotypes Q1 and Affected, the areas under the ROC curves (i.e., the AUC) increased when the phenotypes were adjusted for Age and Smoking status. However, adjusting for these two covariates did not have any influence on the ROC curves for Q2. This is also reasonable because Q2 is not influenced by any covariate, according to the underlying simulation model.

## Discussion

In this study, we evaluated the performance of three methods (four tests)—fixed-threshold, weighted-sum, and variable-threshold methods—for detecting rare variants. The main difference between these methods is the selection of a threshold to discriminate rare from common variants. Based on the simulation model for Q1, most true signals are variants with MAF < 5%, so the T5 test is the best method. The only two exceptions are C13S523 (MAF = 6.67%) in gene *FLT1* and C4S1878 (MAF = 16.50%) in gene *KDR*. However, the powers of the four tests are all high for detecting *FLT1* and *KDR*, because the two genes include many functional variants. Excluding C13S523 from *FLT1* or excluding C4S1878 from *KDR* makes no difference to the final results. The VT test is inferior to the T5 test because of the inclusion of the higher threshold (>5%), which increases noise and reduces power. When analyzing the phenotype Q2, we found that the VT test was the most powerful method for detecting gene *VNN1*. However, both the T1 test and the T5 test performed poorly in detecting *VNN1*, because one of the two functional variants in *VNN1* is relatively common (MAF = 17%). Therefore, when analyzing Q2, the VT test is slightly better than the other methods. For the binary trait (Affected), all tests have similar (poor) performances. This is because the binary trait was determined by a model including noise (Q4). Not surprisingly, analyzing this trait is more challenging than analyzing Q1 or Q2.

Based on our results, we found that inflated type I error rates were caused by potential confounders not adjusted for in the models (Table 1). If a phenotype is related to some covariates, adjusting for these covariates can also increase the true-positive proportions (Q1 and Affected in Figure 1). However, if a phenotype is not related to the covariates, adjusting for them has no influence on the true-positive proportions (Q2 in Figure 1).

## Conclusions

We evaluated the performance of three methods (fixed-threshold, weighted-sum, and variable-threshold methods) in pooling signals of multiple rare variants in a gene. Based on our analyses for the GAW17 data, we find that no method is universally better than the others. Furthermore, adjusting for potential covariates can not only increase the true-positive proportions but also reduce the false-positive proportions. Our study provides an overall evaluation of the three popular pooled association methods with the GAW17 exome simulation data. This can provide insights to determine a strategy for analyzing exome sequencing data.

## Competing interests

The authors declare that they have no competing interests.

## Authors’ contributions

W-YL participated in the design of the study, performed the statistical analysis, and drafted the manuscript. NY, GG, and NL participated in the design of the study and revised the manuscript. BZ participated in the statistical analysis and revised the manuscript. All authors read and approved the final manuscript.
